# Detection of High-Frequency Oscillations by Hybrid Depth Electrodes in Standard Clinical Intracranial EEG Recordings

**DOI:** 10.3389/fneur.2014.00149

**Published:** 2014-08-06

**Authors:** Efstathios D. Kondylis, Thomas A. Wozny, Witold J. Lipski, Alexandra Popescu, Vincent J. DeStefino, Behnaz Esmaeili, Vineet K. Raghu, Anto Bagic, R. Mark Richardson

**Affiliations:** ^1^Brain Modulation Laboratory, Department of Neurological Surgery, University of Pittsburgh, Pittsburgh, PA, USA; ^2^Department of Neurology, University of Pittsburgh Medical Center, Pittsburgh, PA, USA; ^3^Center for the Neural Basis of Cognition, University of Pittsburgh, Pittsburgh, PA, USA; ^4^McGowan Institute for Regenerative Medicine, University of Pittsburgh, Pittsburgh, PA, USA

**Keywords:** epilepsy, high-frequency oscillations, clinical neurophysiology, mesial temporal lobe, ripples, fast ripples

## Abstract

High-frequency oscillations (HFOs) have been proposed as a novel marker for epileptogenic tissue, spurring tremendous research interest into the characterization of these transient events. A wealth of continuously recorded intracranial electroencephalographic (iEEG) data is currently available from patients undergoing invasive monitoring for the surgical treatment of epilepsy. In contrast to data recorded on research-customized recording systems, data from clinical acquisition systems remain an underutilized resource for HFO detection in most centers. The effective and reliable use of this clinically obtained data would be an important advance in the ongoing study of HFOs and their relationship to ictogenesis. The diagnostic utility of HFOs ultimately will be limited by the ability of clinicians to detect these brief, sporadic, and low amplitude events in an electrically noisy clinical environment. Indeed, one of the most significant factors limiting the use of such clinical recordings for research purposes is their low signal to noise ratio, especially in the higher frequency bands. In order to investigate the presence of HFOs in clinical data, we first obtained continuous intracranial recordings in a typical clinical environment using a commercially available, commonly utilized data acquisition system and “off the shelf” hybrid macro-/micro-depth electrodes. These data were then inspected for the presence of HFOs using semi-automated methods and expert manual review. With targeted removal of noise frequency content, HFOs were detected on both macro- and micro-contacts, and preferentially localized to seizure onset zones. HFOs detected by the offline, semi-automated method were also validated in the clinical viewer, demonstrating that (1) this clinical system allows for the visualization of HFOs and (2) with effective signal processing, clinical recordings can yield valuable information for offline analysis.

## Introduction

The surgical treatment for pharmacologically intractable epilepsy stands to offer many patients substantial reduction in seizure burden and even complete seizure freedom (1). This therapeutic approach, although often highly effective, is dependent on the availability of reliable markers for epileptogenic tissue capable of providing clinicians with the spatial information necessary to guide surgical resection. Transient waveforms with spectral content at frequencies above 80 Hz, generally referred to as high-frequency oscillations (HFOs), are observable in human intracranial electroencephalographic (iEEG) recordings (2–5) and a growing body of evidence suggests that these events provide spatial localization information superior to markers currently utilized. Indeed, HFO activity is increased in the primary onset zone (3), better predicts surgical outcomes compared to the clinically identified ictal onset zone (6), and is associated with seizure freedom when its generative tissue is removed (7, 8).

The broad category of HFOs may be subdivided into two distinct subgroups: ripples (R) and fast ripples (FR) are characterized as being dominated by frequency content from approximately 80 to 200 Hz and >250 Hz, respectively. Physiologic HFOs (normal HFOs or nHFOs) in the R band have been recorded in mesial temporal and neocortical structures of non-epileptic animals (9–11) and have been implicated in memory consolidation in humans (12). These nHFOs appear to be mediated by synchronous inhibitory currents onto the perisomatic region of pyramidal neurons serving to temporally coordinate population firing (13, 14). In contrast, FR are not observed in the normal mesial temporal lobe but occur focally following insult and correlate with the frequency of occurrence of resulting seizures (15). These pathologic HFOs (pHFOs) appear to reflect the poorly synchronized firing of subpopulations of pyramidal cells (13) that have escaped the normal inhibitory mechanisms capable of generating R events (16). Given their differences in putative mechanistic underpinnings (see Ref. (17) for review), the ability to map both R and FR events could differentially provide clinicians with invaluable information regarding the spatial extent of epileptic pathology.

With the recent availability of commercial acquisition systems capable of achieving sampling frequencies of 1 kHz and greater, which meet and exceed the Nyquist limit for HFO frequencies, a tremendous amount of data on these novel markers may be generated through standard clinical practice. The utilization of clinically obtained continuous-iEEG recordings, rather than those obtained from specialized research systems, for both continued research and clinical diagnostics would mark a significant advancement in the clinical evaluation of epilepsy; however, factors relating to the type of electrode and acquisition system utilized, quality of recorded signals, and methods for identifying HFO events currently limit the utility of these data. Although numerous studies on HFOs have been conducted, optimal parameters for recording these events have yet to be established and there exists considerable heterogeneity in approaches utilized in the literature.

The nature of recorded events may be influenced by choices in recording techniques. Indeed, studies investigating the spatial distribution of HFOs in humans tend to find that FR events better localize to putative epileptogenic sites when using micro-contacts whereas R events appear to be a better marker when macro-contacts are used (see Ref. (18) for review). Interestingly, direct comparisons of recordings from micro- and macro-contacts demonstrate similar abilities of different contact sizes to detect HFOs with a slight improvement in R detection rates for macro-contacts (5). Further complicating the interpretation of some data, commercially available micro-contacts can vary enormously in their quality and, if not used in conjunction with high impedance acquisition systems, are extremely susceptible to high-frequency noise (19). Finally, an optimal method for detecting HFOs remains to be established. The current gold standard, expert manual review, is extremely time-consuming and may be intractable in light of the volume of data generated by iEEG studies. Numerous automated methods have been proposed, but no one approach has been definitively shown to perform reliably and robustly across data sets.

In order to investigate the ability of expert manual review as well as semi-automated detection methods to identify HFOs in data recorded in a routine clinical setting, we utilized a standard acquisition system sampling at 1 or 2 kHz to record from “off the shelf” hybrid macro-/micro-depth electrodes implanted in the mesial temporal lobe. Recordings were conducted in an unshielded hospital room and were thus susceptible to external sources of noise. Data were then inspected offline for the presence of HFOs by cross-validating (1) manual review using the clinical EEG viewer with (2) a semi-automated approach in which an energy-based detection algorithm identified putative HFOs for subsequent visual validation.

## Materials and Methods

### Subjects

Six patients with pharmacologically intractable focal epilepsy, characterized by focal seizures with alteration of awareness and evolving to a bilateral convulsive seizure, for whom extensive, non-invasive monitoring did not yield concordant data adequate for localizing the region of seizure onset, were included in this study. Following a consensus clinical recommendation from a multidisciplinary epilepsy board, subjects underwent surgery for the implantation of iEEG monitoring in order to localize the seizure onset zone (SOZ). Informed consent for data analysis was obtained from all patients in accordance with a protocol approved by the University of Pittsburgh Institutional Review Board. Five of the six patients underwent unilateral implantation of fronto-temporal subdural grid and strip electrodes in conjunction with simultaneous depth electrode placement in both the amygdala and hippocampus, while one patient underwent the placement of bilateral depth electrodes in the hippocampus.

### Video-EEG recordings

**Figure 1:**
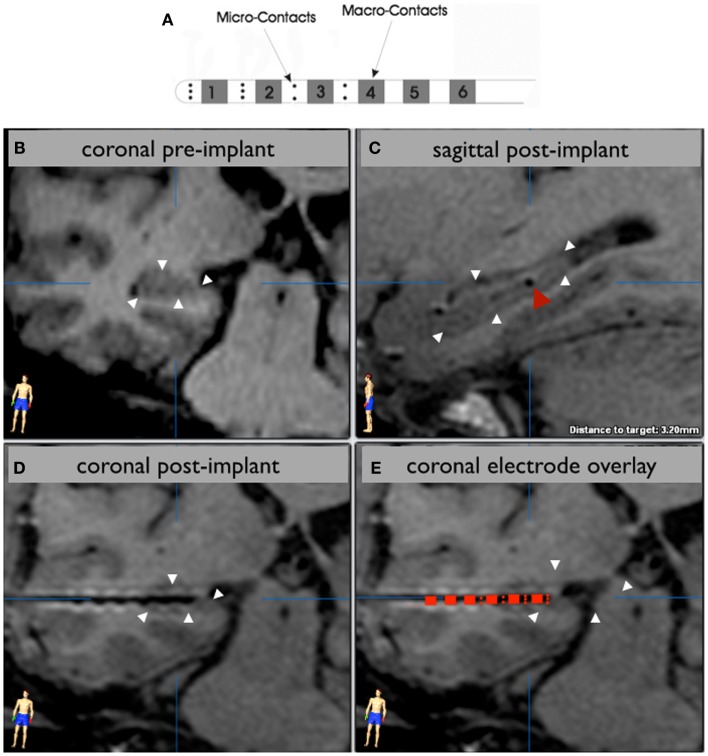
**Electrode localization**. A schematic of the hybrid electrode is shown in **(A)**. The hippocampus is outlined with white arrowheads in a preoperative 3T MRI image **(B)** that corresponds to the in-plane view of an implanted hippocampal depth electrode, visualized by the artifact in the postoperative 1.5 T MRI scan **(C,D)**. A sagittal view **(C)** shows an electrode (red arrowhead) centered in the posterior hippocampal body. A schematic of the electrode is overlaid on the contact artifacts in a magnified coronal view **(E)** to demonstrate which contacts are sampling the hippocampus. In this case, the distal eight micro-contacts and the distal two macro-contacts are within the hippocampus. The distal third macro-contact is located at the lateral border of the hippocampus.

Commercially available, hybrid depth electrodes (Ad-Tech, Inc.) with 10 micro-contacts (50 μm diameter; 0.003 mm^2^ surface area) interspersed between four of the six cylindrical macro-contacts (1.3 mm diameter; 8.88 mm^2^ surface area) were used to collect continuous iEEG recordings from mesial temporal lobe structures (Figure 1A). Continuous iEEG data were recorded in an unshielded hospital room using a 128-channel NATUS Xltech digital video-EEG system (10–50 MΩ amplifier impedance; 1 or 2 kHz sampling rate). Common reference and ground electrodes were placed subdurally at a location distant from any recording electrodes, with contacts oriented toward the dura. Signals were filtered online using a high-pass (0.1 Hz cutoff frequency) and an anti-aliasing low-pass filter. For each patient, overnight recordings were reviewed offline by a clinical neurologist and an approximately 10-min segment of quiet rest or presumed non-REM sleep devoid of seizure activity or amplifier-saturating artifacts was identified for subsequent analysis.

### Electrode localization

The location of electrode contacts was determined using routine postoperative MRI. The artifacts associated with the deepest and most superficial contacts were chosen as the target and entry, respectively, for reformatting the image in the plane of the electrode using BrainLab iPlan software (Figures 1B,C). In this way, each individual contact artifact on a given electrode was visualized simultaneously (Figures 1D,E). Only electrodes verified to be located in the hippocampus or amygdala were analyzed in this study.

### Determination of seizure onset zone

All recorded seizures were visually identified and reviewed by the clinical epilepsy team for each patient. The time of the earliest clear electrographic seizure discharge was selected and marked on the clinical viewer as the electrographic seizure onset time. The location of the macro-contact showing the earliest iEEG change contiguous with seizure onset was used to indicate the SOZ and adjacent micro-contacts were also considered to be within the SOZ.

### Online and offline processing

All online analyses were completed using XLTEK NeuroWorks seven software (Natus Medical Inc., San Carlos, CA, USA) and all offline analyses using MATLAB (MathWorks Inc., Natick, MA, USA) employing a combination of built-in functions, custom routines, and open source code, as described in the following sections.

#### Expert manual review

A board-certified epileptologist visually inspected recordings for the presence of HFOs using the Xltech viewer. All mesial temporal depth electrodes for a given subject were visualized concurrently at a resolution of 120 mm/s. Events were initially marked using a wide-band setting (5 Hz high pass, 60 Hz notch filter, 7–15 μV/mm sensitivity) and subsequently re-reviewed after increasing the high pass to 80 Hz (sensitivity of 3–7 μV/mm). This approach ensured that marked events were apparent in the wideband setting and contained frequency content above 80 Hz. Due to the labor-intensive nature of this review, either 20 temporally discrete events or 1 min of recording, whichever came first, was marked per subject – in this regard, an event co-occurring in spatially adjacent contacts was considered to be one temporally discrete event.

#### “Smart notch” filter

The FFT of each contact’s time-series was computed using the entire duration of the recording to provide an estimate of power content which was maximally resolved in frequency space. A 10 Hz-wide sliding window (2 Hz step size), which scanned each FFT from 100 to 400 Hz, was used to estimate the distribution of local frequency content by computing the median and interquartile range within each window. Power values >8 times the interquartile range above the median were considered to be outliers and represent the local peak in a contaminated frequency band. The width of each contaminated band was then estimated by centering a 10 Hz window on the peak outlier, smoothing this local region of the FFT using a 0.1 Hz-wide moving average, and finding the frequency values on either side of the peak at which the power returned to the median of this new, smoothed window. These aforementioned parameters for detecting contaminated frequency bands were empirically determined by visually inspecting the FFT and detector outputs from every contact using various parameter combinations – the selected parameters identified the majority of user-identified contaminated bands while producing no false positive detections. A set of customized notch filters were then designed for all contaminated frequency bands and run serially on the corresponding time-series in both the forward and reverse directions to minimize phase shifts.

#### Semi-automated detection

An automated detector was modeled after that used in Ref. (20), however, similar to that in Ref. (21), the Hilbert transform was used instead of RMS as a measure of signal energy. Specifically, each contact time-series was notch filtered from 57 to 63 Hz and further de-noised using the smart notch (SN) filter function described above. Notch-filtered signals were then band passed into R- and FR-bands (80–200 and 250–400 Hz, respectively) using a two-way least squares FIR filter designed and implemented using *pop_eegfilt* from the EEGLAB Toolbox (22). The instantaneous amplitude of each band was then computed as the modulus of the Hilbert transform. In order to limit spurious detections resulting from brief, high-amplitude transients, the Hilbert amplitude time-series was smoothed using a 20 ms duration moving average. Upon visual inspection, the distribution of this smoothed amplitude was found to be approximately log-normal, and amplitude values were therefore transformed using the natural logarithm to allow for the use of parametric thresholds. Simulations revealed a minimum amplitude threshold for event detection of three standard deviations greater than the mean to be highly sensitive. The onset and offset of each event exceeding this threshold was marked as the time point when the amplitude fell below two standard deviations above the mean. Detections separated by fewer than 10 ms were considered to be the same event and merged (23).

All putative HFOs were visually validated by two independent reviewers using a custom graphical user interface similar to that described in Ref. (24) and only those events confirmed by both reviewers were retained for subsequent analyses. Reviewers were informed as to whether a given event was detected in either the R- or FR-band but were blind to all other information (SOZ, contact type, diagnosis, etc.). A 1-s window centered on the event was used to simultaneously display the SN-filtered signal, R-band, FR-band, Z-score of R-band, Z-score of FR-band, and spectrogram constructed from the Morlet wavelet decomposition (frequency step size = 6 Hz, σ_frequency_ = 8 Hz) of the SN-filtered signal for frequencies from 50 to 400 Hz. Additionally, a pseudo-FFT depicting the power spectrum specific to the time window of the event was estimated from the wavelet decomposition as the sum of the amplitude within each frequency layer between event onset and offset. The pseudo-FFT for the SN-filtered signal, R-band, and FR-band were overlaid on the same plot for easy comparison and simultaneously displayed with the aforementioned plots. HFOs were considered validated when the following criteria were met: (1) the event did not co-occur with an artifactual transient indicated by a point-to-point voltage change in the SN-filtered signal too large to be of a physiologic origin and/or by broad frequency content spanning the entire HFO band exhibiting a suspiciously geometric (i.e., conical) shape in the spectrogram; (2) there was no evidence of filter ringing as evidenced by power content in the spectrogram specific to filtered frequencies with a symmetric rise and decay profile; (3) the event was clearly discernable from background activity in the notch-filtered signal, its corresponding band-passed signal, and the wavelet decomposition.

### Statistical analyses

Comparisons of the power within contaminated frequency bands were normalized by the median power of the FFT across the HFO frequency band to control for channel differences in global power. Kruskal–Wallis test was used to identify any significant relationships within R- or FR-band events between event detection rate, contact-type (macro- and micro-contacts), and contact location relative to SOZ. Significant relationships were subsequently investigated using a Wilcoxon rank-sum test. All other significant testings were performed using a Wilcoxon rank-sum test. An alpha of 0.05 was used to determine the significance of all statistical test.

## Results

### Localization of seizure onset zone

Five patients were found to have an SOZ in the mesial temporal lobe. One patient had an SOZ in an area of a widespread malformation of cortical development centered in the lateral posterior temporal lobe that also extended into the amygdala and hippocampus. Subject characteristics and the anatomic locations of clinical macroelectrodes associated with the earliest iEEG change at seizure onset are listed in Table 1.

**Table 1:** **Summary of study subjects and clinical data**.

Subject	Age (years)/gender	MRI findings	Electrode locations	SOZ/earliest contacts involved
1	26/F	Normal	Left amygdala and hippocampus	H2/3,A1/2
2	53/F	Normal	Left amygdala and hippocampus	H2/3,A2/3
3	43/F	Left temporal lobe grey matter heterotopia	Left amygdala and hippocampus	PTL
4	24/M	Normal	Right amygdala and hippocampus	H2/3
5	41/F	Normal	Right amygdala and hippocampus	H3
6	24/M	Normal	Right and left hippocampi	Right H1/2

*H, hippocampus; A, amygdala; PTL, posterior temporal lobe*.

### Expert manual review

Concurrently visualizing all depth electrode channels was useful in allowing the reviewer to make observations of overarching trends in the data. All channels of a given contact type (micro or macro) were found to have similar signal quality within a given depth electrode. Furthermore, all macro-contacts were consistently found to have high signal quality across all depth electrodes, which made it possible to observe HFOs in the wide-band signal. In contrast, micro-contacts from 5 of the 11 depth electrodes yielded recordings of an inferior quality leading to their exclusion from this segment of analysis. However, micro-contacts on the six remaining depth electrodes provided recordings which were qualitatively similar to that of adjacent macro-contacts, albeit of lower amplitude.

**Figure 2:**
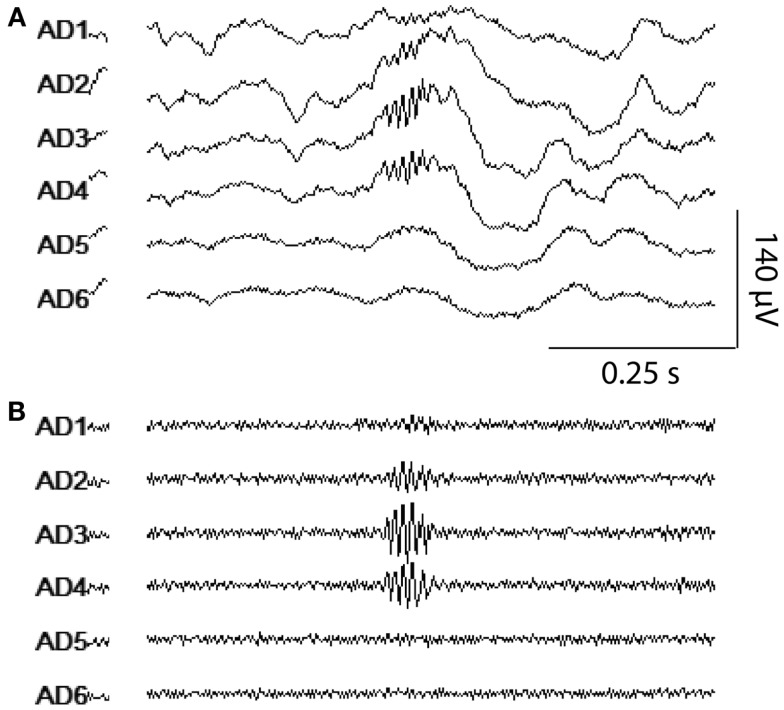
**HFO visualization using the clinical viewer**. A representative example of an HFO detected in multiple macro-contacts simultaneously is shown. Six adjacent macro-contacts from one amygdalar depth electrode were displayed using the clinical viewer software with wide-band [**(A)**; 5 Hz high pass, 60 Hz notch] and HFO-band settings [**(B)**; 80 Hz high pass, 60 Hz notch].

Transient HFOs were identified in all subjects and one subject (subject #4) additionally and uniquely exhibited continuous high-frequency activity throughout the duration of the segment analyzed. This continuous fast activity was observed most strongly in one hippocampal macro and occurred at a lower amplitude in one other neighboring hippocampal macro – both contacts were identified as the SOZ through clinical evaluation. HFOs were observed on as few as one or as many as three adjacent macro-contacts concurrently. It was commonly noted that when events were detected on multiple adjacent macros, the approximate timing of event onset, peak amplitude, and offset as well as the event’s qualitative wave structure was largely consistent across contacts – only the maximum amplitude of the event differed appreciably across contacts (Figure 2). These observations are consistent with the interpretation that one generator was responsible for these events, the activity of which propagated either via the local neuronal circuit or the extracellular fluid to neighboring contacts. Interestingly, events spanning multiple macros were additionally and differentially detected in the spatially intervening micro-contacts. Specifically, events recorded on micro-contacts were generally of lower amplitude compared to adjacent macros, and were often detected on only a subset of micro-contacts at a given level of the depth electrode. When events were detected on two levels of micro-contacts, only micro-contacts located on one side of the depth electrode recorded events suggesting that additional spatial information may be gleaned from radially arranged contacts sampling a smaller area as compared to larger contacts summing activity across the entire circumference of the depth electrode.

### Smart notch filter performance

Visual inspection of recorded time-series and their corresponding FFT plots revealed narrow-band high-frequency noise that was stationary across the duration of recordings in all channels, but particularly pronounced in micro-contacts. Power line noise was commonly observed at 60 Hz and its harmonics as well as contamination at other frequencies which were variable across recording sessions. Preliminary testing showed that this high-frequency contamination in the HFO bands led to unreliable performance of our energy-based HFO detector. In order to mitigate these sources of noise while preserving as much non-contaminated frequency information as possible, we designed a directed filtering routine, the SN filter, capable of identifying and removing narrow-band frequency contamination and implemented it on a per contact basis.

**Figure 3:**
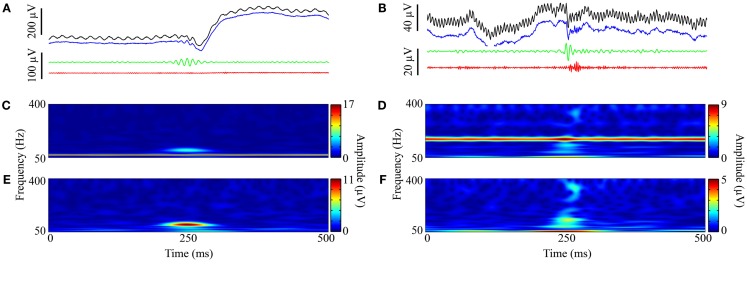
**Representative HFOs and SN filtering performance**. Representative examples showing a ripple **(A,C,E)** and fast ripple **(B,D,F)**. **(A)** and **(B)** each depict four visualizations of an HFO taken from the graphical user interface used for the validation of semi-automatically detected events: (1) unfiltered EEG signal (black), (2) SN-filtered signal (blue), (3) SN and ripple-band passed (80–200 Hz) signal (green), and (4) SN and fast ripple-band passed (250–450 Hz) signal (red). For both **(A)** and **(B)**, the y-axis of black and blue traces are scaled separately from green and red traces. **(C,D)** and **(E,F)** are the spectrograms of the corresponding unfiltered signal (black) and SN-filtered signal (blue), respectively.

**Figure 4:**
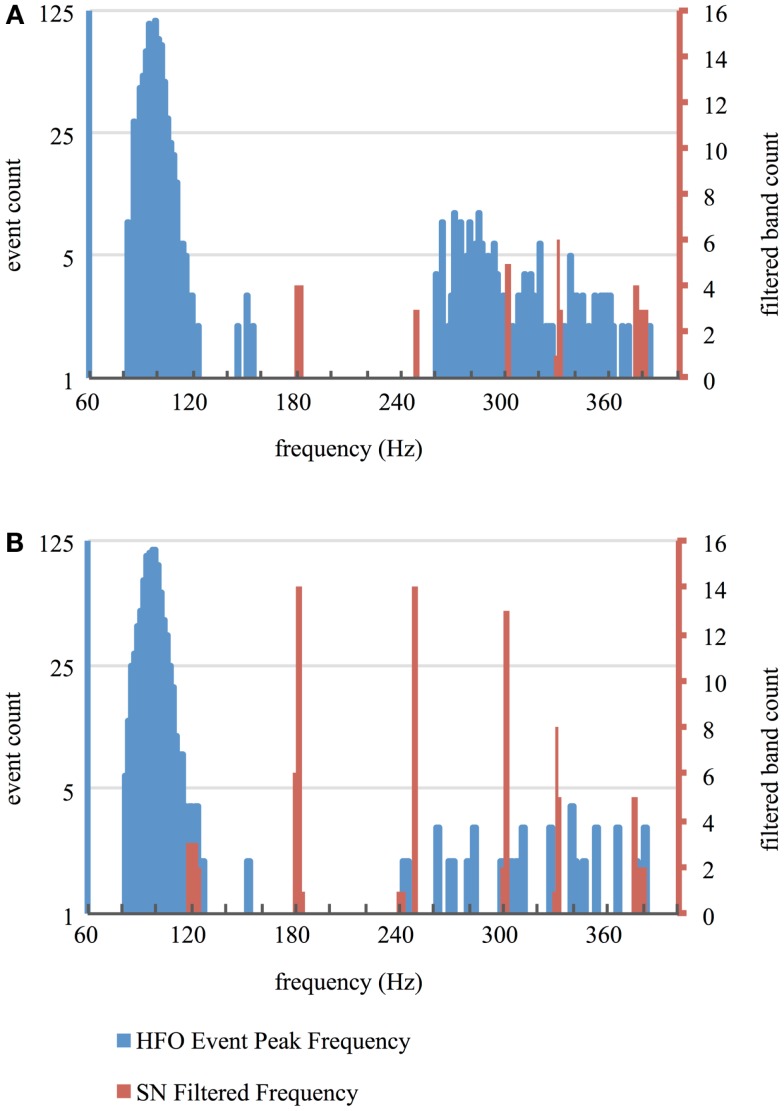
**Frequency of HFOs and SN filtering**. Histograms represent the distribution of peak frequency content of HFOs validated from the entire duration of recording segments using semi-automated methods (blue) as well as frequency bands removed using the SN filter (red) for macro-contacts **(A)** and micro-contacts **(B)**.

Recordings on all contacts contained artifactual frequency bands that commonly occurred at multiples of 60 Hz. Most identified frequency bands were narrow (median width 1.03 Hz; 10^th^ percentile 0.19, 90^th^ percentile 5.18) and, even when taken cumulatively, accounted for a small percentage of the total HFO bandwidth (median 1.9%; 10^th^ percentile 0.5%, 90^th^ percentile 4.9%). The cumulative sum of frequency bandwidths of contaminated frequency bands did not differ between macro and micro-contacts (Wilcoxon, *p* = 0.53), suggesting that similar frequency information remained intact between both contact types, but the power within contaminated bands was significantly greater in micro-contacts (Wilcoxon, *p* = 6.14 × 10^−20^). Identification and removal of this high-amplitude noise through the use of the SN filter allowed fluctuations in HFO-band power of a physiologic magnitude to trigger our automated detector while leaving the vast majority of genuine HFO frequency content intact (Figure 3). Furthermore, the distribution in the peak frequency of confirmed HFOs did not appear to be affected by the notch filtering of contaminated frequency bands (Figure 4).

### Spatial analysis

**Figure 5:**
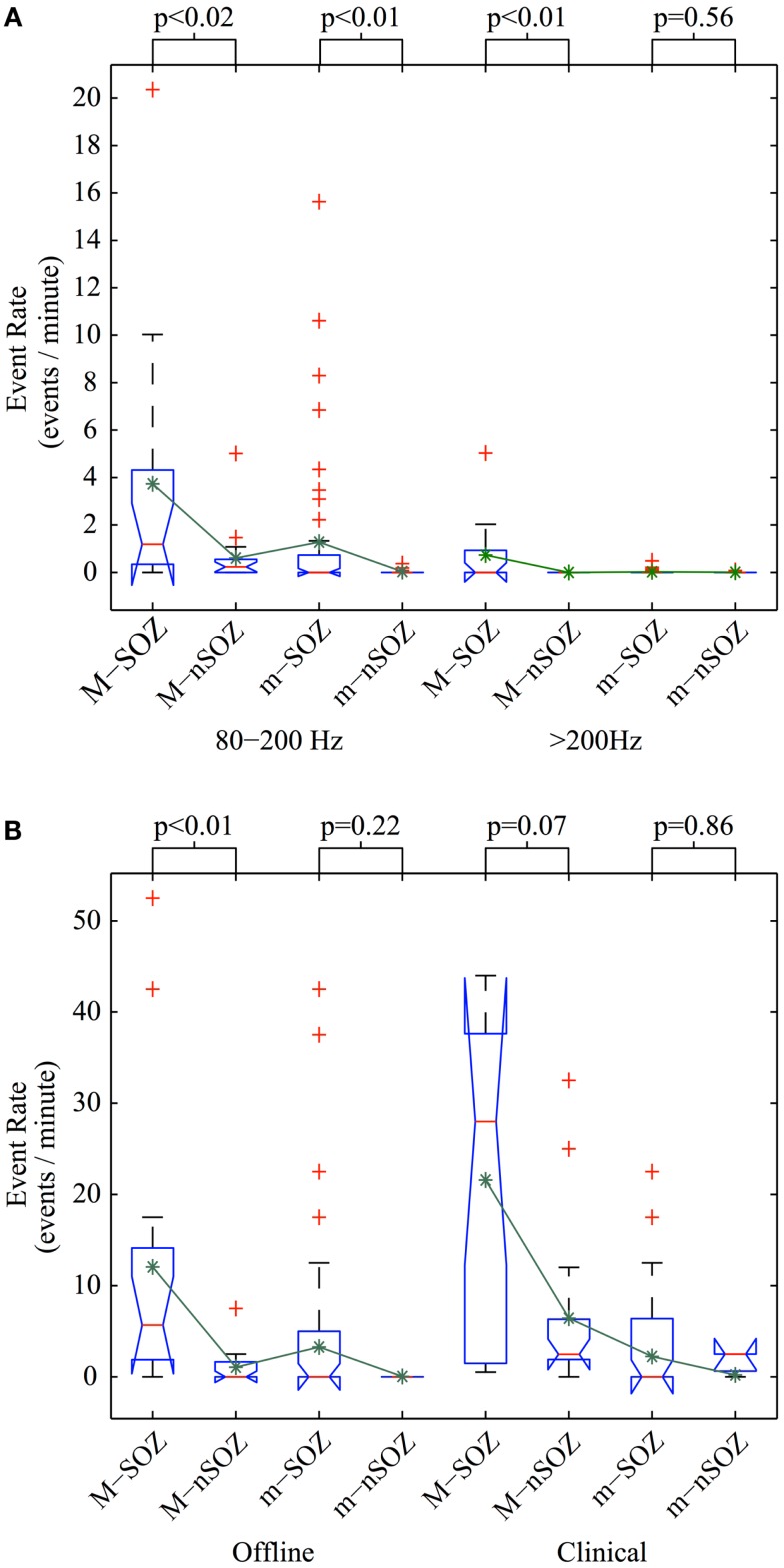
**Comparison of HFO detection rates**. Event detection rate per electrode using **(A)** semi-automated offline methods on full-duration recording segments and **(B)** semi-automated offline methods (left) and manual review (right) for identical recording sub-segments with excluded micro-contacts ignored. Abbreviations used are as follows: M, macro-contact; m, micro-contact; SOZ, seizure onset zone; nSOZ, non-seizure onset zone. Kruskal–Wallis analysis of variance was used to test for significant differences in the mean across three groups: contact type (m vs. M), brain region of interest (SOZ vs. nSOZ), and either HFO frequency range (80–200 vs. >200 Hz in A: test statistic = 71.5, *p* < 0.001) or event detection method (offline vs. clinical in B: test statistic = 30.6, *p* < 0.001). The *p* values for *post hoc* Wilcoxon rank-sum tests are shown above the box plots. Red lines indicate the median; lower and upper borders of blue boxes represent 25^th^ and 75^th^ percentiles, respectively; lower and upper black whiskers correspond to one interquartile range below the 25^th^ and above the 75^th^ percentile, respectively; red pluses depict outliers; and green asterisks show the mean.

An exploratory Kruskal–Wallis test revealed significant differences in the mean detection rates of semi-automatically detected HFOs in both the R- and FR-bands relative to SOZ (Kruskal–Wallis, *p* = 0.00001 and *p* = 0.0009, respectively). For macro-contacts, HFOs occurred more frequently within the SOZ for FR and for R events (Wilcoxon, *p* = 0.0025 and *p* = 0.013, respectively). In contrast, micro-contacts within the SOZ exhibited only a higher R event rate compared to those outside the SOZ (Wilcoxon, *p* = 0.0053) while FR rates did not differ between locations (Wilcoxon, *p* = 0.56). Further inspection of event rates revealed that this latter null result was due to a “floor effect” as very few FR events were identified in micro-contacts at any location. Group-level results for semi-automatically detected HFOs are summarized in Figure 5A. As depicted in Figure 5B, expert manual review revealed a trend level increase in the mean event rate of HFOs in the SOZ for macro-contacts (Wilcoxon, *p* = 0.072) while HFO rates on micro-contacts did not differ by location (Wilcoxon, *p* = 0.86).

To facilitate comparison between event detection methods, both R and FR events detected using semi-automated methods were concatenated into one “HFO” group, and individual events containing frequency content detected in both the R and FR band were further concatenated into one event. Additionally, only events detected during the same time segments on contacts included for inspection by both methods were compared. There was no difference between HFO detection rates in micro-contacts between methods (Wilcoxon, *p* = 0.39), however, significantly higher HFO rates were found in macro-contacts using fully manual methods (Wilcoxon, *p* = 0.012).

## Discussion

In this study, interictal iEEG was recorded from patients with intractable epilepsy in an unshielded hospital room using “off the shelf” hybrid macro-/micro-depth electrodes and a clinical grade acquisition system. This recording setup represents a standard clinical scenario under which iEEG seizure mapping studies are often conducted. The resulting recordings, especially those obtained using micro-contacts, were susceptible to external sources of noise and power line contamination within HFO frequency bands. All macro-contact and approximately half of the micro-contact recordings were of sufficient quality to allow for the identification of HFOs via expert manual review using standard clinical electrophysiology visualization software. We additionally designed an automated, directed filtering routine capable of identifying and removing the powerful noise signals present in these recordings, which enabled an automated event detection algorithm to identifying putative HFOs in all contacts. HFOs visually validated using both detection methods preferentially localized to contact locations within the SOZ, consistent with previous reports.

### Expert manual review using clinical visualization software

In analyzing this clinical dataset, as with any dataset, the importance of understanding the overall quality and global trends present cannot be overstated. In this regard, inspection of these data by means of concurrently visualizing the time-series of all channels of interest was invaluable. The epileptologist reviewing the data was able to quickly assess which channels were and were not usable and to make observations not apparent through semi-automated methods. Namely, the spatial localization of HFOs was readily appreciated as HFO activity clustered in spatially adjacent contacts, with those located centrally in the cluster containing the highest amplitude events. These spatial clusters were remarkably stationary over time and suggestive of a local generator of HFOs. Given that an ideal method for localizing regions of epileptogenic tissue that are both necessary and sufficient for ictogenesis does not currently exist, it is possible that the spatial information gained from concurrent visualization of multichannel data for the identification of HFOs may more accurately circumscribe regions for surgical resection than traditional methods for identifying the SOZ. Indeed, a growing body of evidence suggests that pHFOs may serve as a reliable marker of epileptogenesis. Additionally, the trend-level significance of spatial trends observed in this study may be the result of using short data segments for manual review. While it is likely that using longer duration segments would have improved our power to detect statistical differences, it is remarkable that clear spatial patterns were apparent after inspecting recording segments of only 1-min duration or less. Likewise, it would be preferable to use longer recording segments or even segments from different times (i.e., different nights) to localize HFOs for clinical purposes so as to establish clear and consistent generative locations. As we have demonstrated, these recurring spatial clusters of HFO activity could be visualized by clinicians using standard electrophysiology visualization software – a capability that allows for the use of HFO localization in designing a surgical plan without necessarily incorporating quantitative offline analysis.

Additionally underscoring the benefit of manual review, a unique observation in one patient was made during visual inspection: one macro-contact in the hippocampus contained persistent high-frequency activity throughout the duration of the recording segment, and one neighboring macro-contact in the same depth electrode also contained this continuous activity although at a lower amplitude. Interestingly, these contacts were identified through standard clinical evaluation as being the SOZ. This unique example further demonstrates the utility of simultaneous visual inspection in generating an accurate and reliable interpretation of recordings. In contrast, our semi-automated method differentially identified this activity as numerous separate events, displaying only one putative event at a time for review, and thus did not accurately reflect the spatiotemporal dynamics of the true neural activity.

### Smart notch filtering and recording contamination

The application of the automated, data-driven SN filter to this clinical dataset had several advantages. Notably, because the routine used statistical measures estimated from the local frequency power content of the FFT to define its detection thresholds, global trends in frequency content, such as the well-known spectral roll-off often observed in electrophysiological signals, did not affect the identification of contaminated frequency bands. Furthermore, this initial noise-identification step allowed the user to characterize the quality of a given recording on a per contact basis. When comparing noise contamination from recordings using micro- and macro-contacts, it was apparent that the substantially more powerful noise observed in micro-contacts was not the result of a greater bandwidth of contaminated frequencies but rather more powerful noise within similar frequency bands. This finding suggests that, although the use of micro-contacts in conjunction with a comparatively low impedance acquisition system yields recordings which are highly susceptible to noise [as noted in Ref. (19)], these recordings may still possess a bandwidth of informative frequency content similar to that of macro-contact recordings. Indeed, a 60 Hz notch filter was sufficient to render micro-contacts from 6 of the 11 depth electrodes useful in manual visual review. Additionally and perhaps most important to the integrity of de-noised recordings, the SN filter was highly frequency specific in removing noise by (1) using the FFT amplitude calculated from the entire duration of the recording to maximize frequency resolution when defining the frequency borders of noise bands and (2) by using a notch filter with a narrow stopband. Indeed, the median bandwidth of filtered frequencies was only 1.03 Hz in this dataset. Finally, de-noising using the SN filter routine required a minimal amount of time to “clean” recordings given that it was not only automated, but also computationally inexpensive: the SN filter screened and de-noised the entire HFO band (80–450 Hz) of a 15-min recording segment containing 6 macro- and 10 micro-contacts (1 kHz sampling) at a rate of approximately one channel per second (computation time estimated using an 2009 MacBook Pro with 8 GB of RAM and a dual-core 2.8 GHz processor).

Because a notch filter removes all frequency content within its filter borders, there exists the possibility that some of the frequency content of some HFOs was removed by the filter. However, because the bandwidth of individual noise bands removed was quite narrow (median width = 1.03 Hz), it is unlikely that the frequency content of any given HFO was entirely removed. The spectrograms in Figure 3 corroborate this assumption, as noise-related frequency content is focally removed and much of the HFO-related activity is retained. Furthermore, the vast majority of the HFO bandwidth was unaltered by notch filtering (median percentage of the total HFO bandwidth removed was 1.9%). Consistent with the interpretation that HFO detections were not confounded by the use of the SN filter, the distribution of peak frequency content of visually validated HFOs does not correspond to any contaminated bands removed by notch filtering (Figure 4). It is worth noting that the use of an energy-based automated HFO detection algorithm would not have been possible without the use of SN filter as the variance of noise was so great that it artificially increased the threshold for event detection beyond that which events of physiologic magnitude could exceed. Taken together, these results demonstrate the utility of directed de-noising approaches, such as the SN filter, in inspecting clinical data for the presence of HFOs.

### Semi-automated offline analysis

As the time-intensive nature of manual review is well appreciated, semi-automated methods for HFO detection stand to reduce this user burden while still allowing for human oversight. Our semi-automated approach was indeed effective in identifying HFOs; however, our decision to forgo any automated pre-screening of events placed a considerable demand on human reviewers to validate the immense number of detections (nearly 38,000). Given that the behavior of such a clinical dataset has not yet been well characterized, the choice to only automate the initial step of event detection was made in an attempt to maximize the sensitivity of our analyses and avoid any false negatives resulting from an over-active pre-screening routine. Our results are significant in that they demonstrate the utility of clinical recordings in allowing for robust offline analyses of HFOs. Going forward, the thoughtful application of pre-screening procedures in semi-automated methods will expedite event validation considerably. While the utility of automated and semi-automated methods may not be fully realized in direct clinical application – the clinical viewer may readily provide a heuristic of HFO localization to the experienced reviewer – the finely resolved spatial and temporal output of such approaches is invaluable in a research setting for the exploration of event-related activity, connectivity, and other such analyses. After analyzing the same dataset with two separate detection methods, it seems that the most robust method for the offline identification of HFOs would be a semi-automated method capable of automatically detecting and pre-screening events for subsequent visual validation using a multi-channel display. By displaying multi-channel data with automatically detected events highlighted for review, user time would be dramatically reduced not only through automatic detection but also by allowing the user to validate many channels simultaneously. This approach would still allow for and help to optimize human supervision by allowing the individual to appreciate overarching trends in the data as well as easily inspect the spatiotemporal context in which events occur.

### Influence of contact type

The use of hybrid depth electrodes in this study additionally allowed for the direct comparison of recordings obtained with macro and micro-contacts. Even though recordings were conducted in an unshielded clinical environment, macro-contacts were resistant to external sources of noise aside from very narrow frequency-band contamination. This favorable data quality was apparent during manual review when HFO activity was clearly discernable after the application of only a 60 Hz notch filter. Recordings from micro-contacts were markedly more susceptible to noise and more variably affected across recording segments – a 60 Hz notch filter was sufficient for de-noising micro-contacts from 6 of the 11 depth electrodes while the remaining 5 required more thorough de-noising with the SN filter before HFOs could be observed. As noted in Ref. (19), high impedance micro-contacts are extremely sensitive to noise when used in conjunction with relatively low impedance acquisition systems such as the clinical system applied in this study. Our observations are consistent with this previous report and, although targeted de-noising may greatly improve the signal-to-noise ratio of recordings, we do not recommend using low-impedance systems to record from micro-contacts.

An intriguing observation regarding the distribution of HFO activity across individual depth electrodes was made during manual review: the radial arrangement of micro-contacts differentially recorded HFOs even within a given level of the depth electrode. Because macro-contacts spanned the entire circumference of the depth electrode, activity from all sides was summed and directional information was thus lost. However, the focal spatial sampling of micro-contacts allowed for the differentiation of activity from neighboring patches of tissue. In this case, observing an HFO in all micro-contacts at a given level of the depth electrode would suggest that the electrode is located somewhere *within* the generative tissue but observing an HFO in only one micro-contact would suggest that the electrode may lie at the lateral border of the neuronal ensemble responsible. This observation underscores the profound influence that electrode construction has on the nature of recordings and further indicates that improvements in electrode design may enable clinicians and researchers to better localize electrophysiological events within the brain.

### Limitations

This study analyzed data from a modest number of patients and conclusions drawn from these results should be made with this fact in mind. Another caveat in the interpretation of our results exists in regard to the location of ictogenesis: five out of the six patients included in this study had a SOZ in the mesial temporal lobe. Although this location constitutes the most common site of seizure onset, neocortical and extratemporal lobe epilepsy was not considered in this study. Characteristics of HFOs may vary by brain region and this variability may interact with electrode type to influence the nature of recorded events. For example, it is not known precisely how the spatial extent of networks responsible for generating HFOs varies by brain region and the size of these networks may differentially affect how they are recorded with macro versus micro-contacts. In order to address these issues, the described methods will be applied to a large cohort of subjects having both temporal and extratemporal onset zones in a future study.

## Conclusion

This study demonstrates that iEEG recordings obtained on a clinical grade recording system are useful in the identification of HFOs in both research and clinical setting; however, special care should be taken into control for noise encountered in these recordings. Furthermore, the use of micro-contacts should be limited to high-impedance systems but, if so arranged, may provide added spatial information. Additionally, although both manual review using clinical software and semi-automated detection are capable of identifying HFOs in clinical recordings, concurrently visualizing activity from numerous channels more accurately depicts neural activity and is invaluable in helping the reviewer to hone the internal criteria used for distinguishing genuine HFOs. The development of multichannel methods for visually validating automatically detected HFOs in a rapid and robust manner would be beneficial. To this end, we are developing a graphical user-interface which plots multi-channel data with automatically detected events highlighted and allows the reviewer to quickly add/remove detections as well as plot additional information such as wavelet-based spectrograms. Finally, making the analytic codes developed for such studies freely available, as we have done here with our SN filter routine (available from authors upon request), may assist groups in standardizing detection and analytic approaches in HFO research.

## Conflict of Interest Statement

The authors declare that the research was conducted in the absence of any commercial or financial relationships that could be construed as a potential conflict of interest.
